# IDENTIFYING TOOLS TO MEASURE NURSING HOME RESIDENT GOALS, PRIORITIES, AND PREFERENCES FOR PERSON-CENTERED CARE

**DOI:** 10.1093/geroni/igae098.1217

**Published:** 2024-12-31

**Authors:** Tonya Roberts, Romil Parikh, Jingxi Li, Megan Nizza, Amelia Smith, Sorah Levy, Tetyana Shippee

**Affiliations:** University of Wisconsin - Madison, Madison, Wisconsin, United States; University of Minnesota, Minneapolis, Minnesota, United States; University of Wisconsin Madison, Madison, Wisconsin, United States; Boston University, Boston, Massachusetts, United States; Georgetown University, Washington, District of Columbia, United States; University of Maryland School of Nursing, University of Maryland School of Nursing, Maryland, United States; University of Minnesota School Of Public Health, Minneapolis, Minnesota, United States

## Abstract

Identifying nursing home (NH) residents’ goals, priorities, and preferences is a critical step in the delivery of person-centered care. While numerous instruments and tools exist to measure resident preferences, it remains unclear how psychometrically validated instruments used in research may be feasibly applied in practice. In this review, we aimed to identify instruments for assessing residents’ goals, priorities, and preferences and develop recommendations for application in practice. We searched MEDLINE, CINAHL, and PsycINFO databases to identify instruments that assessed quality of life, satisfaction, person-centered care, psychosocial needs and goals, and preferences and were validated in US NHs. We also surveyed experts in the field to identify assessment tools from grey literature. We identified a total of 28 validated tools from 23 peer-reviewed articles and 12 additional tools from 35 grey literature sources. These tools varied in structure (e.g. open vs closed questions), content (e.g. domains covered), and length (e.g. few to many items). Tools could be grouped into two major types: generative and evaluative. Generative tools assessed specific resident goals, priorities, and preferences that could inform care planning particularly upon admission, but also throughout the resident’s stay. Evaluative tools required residents to evaluate aspects of care and may be most appropriate beyond admission. NH staff may find different benefits to various assessment approaches and may need to make local decisions about which to use and which will integrate into existing workflows effectively.

